# Influence of Cardiovascular Risk Burden on Motor Function Among Older Adults: Mediating Role of Cardiovascular Diseases Accumulation and Cognitive Decline

**DOI:** 10.3389/fmed.2022.856260

**Published:** 2022-04-26

**Authors:** Zhangyu Wang, Kaiwang Cui, Ruixue Song, Xuerui Li, Xiuying Qi, Aron S. Buchman, David A. Bennett, Weili Xu

**Affiliations:** ^1^Department of Epidemiology and Biostatistics, School of Public Health, Tianjin Medical University, Tianjin, China; ^2^Tianjin Key Laboratory of Environment, Nutrition and Public Health, Tianjin, China; ^3^Center for International Collaborative Research on Environment, Nutrition and Public Health, Tianjin, China; ^4^Department of Respiratory and Critical Care Medicine, The Fifth People's Hospital of Ganzhou, Ganzhou Institute of Respiratory Diseases, Ganzhou, China; ^5^Shandong Provincial Clinical Research Center for Emergency and Critical Care Medicine, Institute of Emergency and Critical Care Medicine of Shandong University, Qilu Hospital of Shandong University, Jinan, China; ^6^Rush Alzheimer's Disease Center, Rush University Medical Center, Chicago, IL, United States; ^7^Aging Research Center, Department of Neurobiology, Care Sciences and Society, Karolinska Institutet, Stockholm, Sweden

**Keywords:** Framingham general cardiovascular risk score, motor function, cardiovascular diseases, cognitive decline, cohort study

## Abstract

**Purpose:**

This study aimed to investigate the association of the cardiovascular risk burden assessed by the Framingham General Cardiovascular Risk Score (FGCRS) with the trajectories of motor function over time and to assess the mediating effects of cardiovascular diseases (CVDs) accumulation and cognitive decline in such association.

**Methods:**

In Rush Memory and Aging Project, a total of 1,378 physical health participants (mean age: 79.3 ± 7.3 years) were followed up for up to 22 years. FGCRS at baseline was assessed and categorized into tertiles (lowest, middle, and highest). Global motor function (including dexterity, gait, and hand strength) was assessed annually with 10 motor tests. CVDs (including stroke, congestive heart failure, and other heart diseases) were ascertained at baseline and follow-ups, and the number of CVDs accumulation over time was assessed. Global cognitive function was tested annually by 19 tests. Data were analyzed using the linear mixed-effects models and mediation analysis.

**Results:**

At baseline, FGCRS ranged from 4 to 28 (mean score: 15.6 ± 3.7). Over the follow-up (median: 5.3 years; interquartile range: 2.9–9.0 years), in multi-adjusted mixed-effects models, the highest FGCRS was associated with faster decline in global motor function (β = −0.0038; 95% confidence interval [CI]: −0.0069 to −0.0008), dexterity (β = −0.0056; 95% CI: −0.0093 to −0.0020), gait (β = −0.0039; 95% CI: −0.0077 to −0.0001), and hand strength (β = −0.0053; 95% CI: −0.0098 to −0.0008) compared with the lowest tertile. In mediation analysis, CVDs accumulation and cognitive decline mediated 8.4% and 42.9% of the association between FGCRS and global motor function over time, respectively.

**Conclusion:**

Higher cardiovascular risk burden is associated with a faster decline in motor function including dexterity, gait, and hand strength. CVDs accumulation and cognitive decline may partially mediate the association between cardiovascular risk burden and global motor function decline.

## Introduction

Population aging is accelerating worldwide, while the size of the elderly population and its share of the total population are growing rapidly (1). Meanwhile, motor functions such as dexterity, mobility, coordination, and muscle strength will decline gradually with increased age in the elderly. Motor function decline has been shown to be a precursor to disability and an independent predictor of death (2), which have added extra growing costs to the already strained health- and social-care systems (3). Therefore, timely preventive strategies to monitor motor function decline are essential to control the occurrence and progression of disability to improve the quality of life in older adults.

A number of studies have demonstrated that single cardiovascular risk factors, such as smoking (4), hypertension (5), diabetes (6), and hyperlipidemia (7), were associated with decreased motor function in the elderly. Although interventions for single cardiovascular risk seem to be easier to implement, these cardiovascular risk factors tend to cluster and interrelate so that most older adults often suffer from two or more vascular disorders (8). The Framingham General Cardiovascular Risk Score (FGCRS) is a risk algorithm that combines demographics with traditional cardiovascular risk factors to assess the burden of cardiovascular disease (CVD), allowing for integrated prediction of CVD risk in individuals (9). So far, only two studies have explored the relationship between FGCRS and motor function in older adults previously, which suggested that higher FGCRS was associated with poorer performances in walking speed (10, 11). However, the impact of vascular risk burden on trajectories of global and specific motor functions remains unclear.

Many studies have shown that people with higher FGCRS are at increased risk of CVDs (12, 13) and cognitive decline (14, 15), which are associated with motor function impairments in older age (16–19). However, no studies have assessed whether and to what extent CVDs accumulation and cognitive decline may mediate the association between FGCRS and motor function. In this study, we aimed to examine the association between FGCRS and trajectories of global and specific motor functions and to explore the mediating role of CVDs accumulation and cognitive decline in this association using 22 years of follow-up data from a community-based cohort study.

## Materials and Methods

### Study Population

The Rush Memory and Aging Project (MAP) is an ongoing longitudinal prospective cohort study on the decline in cognition and motor function in the elderly. The MAP study's design and evaluation protocol were described in detail previously (20). In brief, participants were recruited in northeastern Illinois from continuous care retirement communities, religious organizations, senior and subsidized housing, and social assistance agencies. A total of 2,192 participants were tracked annually for up to 22 years from 1997 to 2020.

Of all participants, 814 participants including 573 participants with missing data on FGCRS, 260 participants with a disability in basic activities of daily living (ADL) at study entry, and 462 participants with missing data on global motor function at baseline or during follow-up were excluded. Finally, a total of 1,378 participants were admitted to this study (Supplementary Figure 1).

Rush Memory and Aging Project received approval from the Rush University Medical Center's Institutional Review Board (21). In accordance with the ethical standards set out in the 1964 Declaration of Helsinki and its subsequent amendments, all participants signed informed consent and a repository consent at baseline to allow their data to be shared after being informed in detail of the risks and benefits of participating in the study. The Rush Alzheimer's Disease Center Resource Sharing Hub may be accessed at https://www.radc.rush.edu for more information on the data.

### Data Collection

Each participant obtained a uniform comprehensive clinical evaluation including detailed medical histories, thorough physical examination, and systematic function tests by trained staff at the time of enrollment and follow-up (22). Data on demographic characteristics, socioeconomic status, anthropometric indicators, and daily lifestyle factors were also securable at baseline and at each follow-up visit.

Education level was obtained based on the maximum years of regular schooling (range: 3–28 years) (23). Alcohol consumption was measured by the total grams of alcohol a participant consumed per day in the past year. Smoking was divided into three categories, namely, never smoker, former smoker, and current smoker. Physical activity was generated from the total number of hours per week that participants engaged in 5 items of activities according to the 1985 National Health Interview Survey (24). Social activity in later life was assessed by a six-item scale of the frequency of participation in common types of social interaction activities over the past year, with higher scores indicating more social participation (25). Height and weight were measured and documented by a trained technician who was unaware of the previously collected data (26). Body mass index (BMI) was calculated by dividing weight in kilograms by the square of height in meters.

Participants sat with their arms at heart level and had their blood pressure measured two times at 5-min intervals on their right arm by trained research assistants using a mercury sphygmomanometer, and the average of the two measurements was recorded as the blood pressure value. Hypertension was considered as systolic blood pressure (SBP) ≥140 mm Hg, diastolic blood pressure (DBP) ≥90 mm Hg, or the use of antihypertensive medications.

Blood samples were taken from all the participants, and total cholesterol (TC), high-density lipoprotein (HDL) cholesterol, and blood glucose levels were measured by a professional diagnostic company (22). Diabetes was defined as the presence of any of the following criteria: hemoglobin A1c in blood samples ≥6.5%, fasting plasma glucose ≥126 mg/dl, random blood glucose ≥200 mg/dl, history of diabetes, or use of diabetes medications (27).

Activities of daily living was a composite measure of disability using the Katz ADL Scale, which measured six basic physical abilities, namely, eating, dressing, bathing, toileting, getting from bed to chair, and walking across a small room (28).

### Assessment of FGCRS

Following the Framingham General Cardiovascular Risk Prediction Model, we assessed and calculated the FGCRS at baseline for each participant based on the information on age, sex, current smoking status, SBP, antihypertensive medication use, diabetes mellitus, TC, and HDL cholesterol (Supplementary Tables 1, 2) (9). All these risk factor scores were summed to obtain the FGCRS, and the scores were further categorized into the lowest, middle, and highest tertiles. A higher FGCRS indicated an increased risk of future cardiovascular events.

### Assessment of CVDs

Cardiovascular diseases encompassed congestive heart failure, stroke, and other heart diseases including heart attack or coronary, coronary thrombosis, coronary occlusion, and myocardial infarction. Stroke events were obtained based on a clinician review of self-reported questions, neurological examination (if available), cognitive testing, and interviews with participants. Information on congestive heart failure was obtained from self-reports by asking participants if they had been told that they had congestive heart failure since the date of the last interview by a doctor, nurse, or therapist. Information related to other heart diseases was obtained in the same manner as above except for the questions revolved. The three categories of stroke, congestive heart failure, and other heart diseases were dichotomized as present and absent at baseline and follow-ups, and CVD events accumulation was summed as the number of CVDs (range: 0–3).

### Assessment of Global Cognitive Function

A battery of 21 cognitive performance tests was used to assess cognitive function at baseline and at each follow-up. Of the 21 tests, the Mini-Mental State Examination (MMSE) was used primarily to describe the cohort but not for analysis, while another test named Complex Ideational Material was only used for diagnostic classification. The remaining 19 tests, namely, Word List Memory, Word List Recall, Word List Recognition, immediate and delayed recall of the East Boston Story and Story A from Logical Memory, Boston Naming Test, Category Fluency, National Adult Reading Test, Digit Span Forward and Backward, Digit Ordering, Symbol Digit Modalities Test, Number Comparison, word reading and color naming from a modified version of the Stroop Neuropsychological Screening Test, Judgment of Line Orientation, and the Standard Progressive Matrices, were used to construct a summary measure of global cognitive function (22).

The raw scores of each cognitive performance test were converted to *z-*scores, which were averaged to yield a global cognition score if more than half of the *z-*scores were not missing. The score describes the extent to which a point deviates from the mean or a specific point, while higher global cognition scores indicate better global cognitive function.

### Assessment of Motor Function

A comprehensive measure of global motor function including subcomponents of dexterity, gait, and hand strength was evaluated by a 10-item motor function test at baseline and at each follow-up visit.

*Motor dexterity* is a combined measure consisting of two motor tests, namely, the Purdue pegboard test and the finger-tapping test. Participants were asked to place pegs into the holes of a pegboard (Purdue Pegboard) within 30 s and to tap an electronic tapper with their index finger as fast as possible for 10 s. The above trials were performed two times for each hand separately. The average of the number of pegs placed into the pegboard and the number of taps by each participant in four trials yielded a Purdue Pegboard score and a tapping score, respectively.

To evaluate *motor gait*, we asked participants to walk a distance of 8 feet two times and turn 360° two times, recording the time and number of steps required for each test. The two measurements for each test were averaged to provide the following performance scores: walking time, walking steps, turning time, and turning steps.

*Hand strength* was composed of two tests, namely, grip strength and pinch strength. We measured participants' grip and pinch strength two times for each hand using the Jamar hydraulic hand and pinch dynamometers (Lafayette Instruments, Indiana). The average of the four trials for each test represented the performance scores for grip and pinch strength in terms of pounds of pressure. *Balance* was assessed by asking participants to stand on each leg and their toes for 10 s each.

The *raw scores for each of the above 10 tests* were converted to *z-*scores, and the results of all motor tests were averaged together to construct a global motor function score.

### Statistical Analysis

To compare the differences of the baseline characteristics among three groups of participants by FGCRS tertiles, one-way ANOVA or Kruskal-Wallis rank-sum test was used for numerical variables, and the chi-square test was used for categorical variables.

The β-coefficients and 95% confidence intervals (CIs) for the association between cardiovascular risk burden (i.e., continuous and categorical FGCRS) and annual changes in global motor function and the three specific motor functions were estimated using linear mixed-effects models with follow-up time (years) as the time scale. The fixed effect included cardiovascular risk burden, follow-up time, and their interactions. The random effect included random slopes and intercepts, allowing for the individual differences at baseline and across follow-up.

To test and quantify the mediating role of CVDs accumulation and cognitive decline in the association between FGCRS and changes in motor function, we performed a mediation analysis in two independent models using the bootstrapping methods. Linear mixed-effects models were used to test the three pathways of the mediation analysis: (1) the association between FGCRS and changes in motor function (total effects); (2) the association between FGCRS and CVDs accumulation or cognitive decline; and (3) the association between CVDs accumulation or cognitive decline and changes in motor function, controlling for FGCRS. The mediating role was significant if the bias-corrected 95% CI of the indirect effects estimated by bootstrapping did not include zero (29).

Education, BMI, alcohol consumption, physical activity, social activity, baseline number of CVDs, and baseline global cognitive function as potential confounders were adjusted in the linear mixed-effects models and mediation analysis.

In the sensitivity analysis, we excluded 382 participants who had dementia at baseline and during the follow-up because some components of FGCRS and CVDs were based on self-report. Linear mixed-effects models and mediation analysis were repeated in the above populations severally.

A two-tailed *P*-value < 0.05 was considered statistically significant for each test. All statistical analyses were performed using Stata SE 15.0 (Stata Corp LP., College Station, Texas, United States).

## Results

### Characteristics of the Study Population at Baseline

Among all participants (*n* = 1,378; mean age: 79.3 ± 7.3 years; 73.5% female participants), FGCRS ranged from 4 to 28 (mean score: 15.6± 3.7) at baseline, and 530 (38.5%) participants had the lowest FGCRS, 412 (29.9%) participants had middle tertile, and 436 (31.6%) participants had the highest tertile.

Compared with participants in the lowest FGCRS, those with the highest FGCRS were more likely to be older, male, and smoker and have higher BMI, diabetes, hypertension, and CVDs, but lower level of education, physical activity, social activity, HDL, and global cognitive function, as well as poorer global motor function and motor dexterity at baseline. There were no significant differences in terms of alcohol consumption, TC, follow-up time, motor gait, and motor hand strength among the three groups (Table 1).

**Table 1:** Baseline characteristics of the study population by Framingham General Cardiovascular Risk Score (FGCRS) categories (*n* = 1,378)^*^.

	**FGCRS (in tertiles)**			
**Characteristics**	**Lowest *n =* 530 (38.5%)**	**Middle** ***n =* 412** **(29.9%)**	**Highest *n =* 436 (31.6%)**	***P-*value**
Age (years)	77.05 ± 8.21	80.62 ± 6.36	80.82 ± 6.14	<0.001
Female, *n* (%)	465 (87.7)	296 (71.8)	252 (57.8)	<0.001
Education (years)	15.45 ± 3.08	14.95 ± 3.22	14.97 ± 3.18	0.020
Body mass index (kg/m^2^)	26.40 ± 4.80	27.27 ± 5.52	28.15 ± 4.74	<0.001
Alcohol consumption (g/d), median(IQR)	1.08 (0.00,7.00)	1.08 (0.00,7.10)	0.00 (0.00,6.04)	0.640
Physical activity (h/w), median(IQR)	3.00 (1.17,5.44)	2.79 (1.17,4.75)	2.50 (0.75,4.66)	0.019
Social activity, median (IQR)	2.83 (2.50,3.17)	2.67 (2.33,3.00)	2.67 (2.20,3.00)	<0.001
Smoking status, *n* (%)				0.041
Never	310 (58.5)	253 (61.4)	243 (55.7)	
Ever smoker	212 (40.0)	150 (36.4)	174 (39.9)	
Current smoker	8 (1.5)	9 (2.2)	19 (4.4)	
TC (mg/dL)	191.36 ± 33.98	194.66 ± 41.16	196.08 ± 45.09	0.215
HDL (mg/dL)	66.23 ± 16.88	60.24 ± 17.63	53.44± 16.61	<0.001
SBP (mmHg)	123.01 ± 12.80	135.59 ± 14.38	147.92 ± 16.59	<0.001
FGCRS	11.84 ± 2.09	15.98 ± 0.81	19.77 ± 1.77	<0.001
Diabetes, *n* (%)	19 (3.6)	31 (7.5)	125 (28.7)	<0.001
Hypertension, *n* (%)	256 (48.3)	318 (77.2)	417 (95.6)	<0.001
CVD, *n* (%)	63 (11.9)	72 (17.5)	93 (21.3)	<0.001
MMSE	28.49 ± 1.67	27.80 ± 2.60	27.76 ± 2.42	<0.001
Global cognitive function, median (IQR)	0.31 (−0.08,0.60)	0.13 (−0.29,0.47)	0.07 (−0.35,0.41)	<0.001
Follow-up time (years), median (IQR)	5.98 (2.91,9.60)	5.37 (2.95,9.06)	5.03 (2.97,8.97)	0.331
Global motor function	1.09 ± 0.22	1.03 ± 0.21	1.02 ± 0.20	<0.001
Motor dexterity	1.07 ± 0.16	1.02 ± 0.13	1.00 ± 0.15	<0.001
Motor gait	1.07 ± 0.25	1.05 ± 0.25	1.05 ± 0.25	0.271
Motor hand strength	1.06 ± 0.33	1.02 ± 0.29	1.05 ± 0.29	0.102

* *Values are means±SDs unless otherwise indicated.*

*Missing data: Body mass index = 6, motor hand strength = 66.*

*BMI, body mass index; TC, total cholesterol; HDL, high-density lipoprotein; SBP, systolic blood pressure; FGCRS, Framingham General Cardiovascular Risk Score; CVD, cardiovascular disease*.

### Relationship Between FGCRS and Motor Function Decline

At baseline, FGCRS was associated with global motor function (β = −0.0048; 95% CI: −0.0076 to −0.0020) and motor dexterity (β = −0.0049; 95% CI: −0.0069 to −0.0029) when FGCRS was used as a continuous variable. When FGCRS was used as tertiles, participants with the highest tertile had poorer motor dexterity (β = −0.0356; 95% CI: −0.0533 to −0.0178) compared to those with the lowest tertile (Table 2).

**Table 2:** β-coefficients and 95% confidence intervals (CIs) for the association of the Framingham General Cardiovascular Risk Score (FGCRS) with the changes of global and specific motor functions over time: results from mixed-effect models^*^.

**Cardiovascular disease risk**	**Global motor function**	**Motor dexterity**	**Motor gait**	**Motor hand strength**
**Baseline**				
Continuous FGCRS	−0.0048^†^ (−0.0076 to −0.0020)	−0.0049^†^ (−0.0069 to −0.0029)	0.0001 (−0.0031 to 0.0033)	−0.0011 (−0.0051 to 0.0028)
**FGCRS categories**				
Lowest risk	Reference	Reference	Reference	Reference
Middle risk	−0.0305^†^ (−0.0549 to −0.0061)	−0.0198^†^ (−0.0374 to −0.0022)	−0.0069 (−0.0352 to 0.0213)	−0.0141 (−0.0487 to 0.0204)
Highest risk	−0.0229 (−0.0475 to 0.0017)	−0.0356^†^ (−0.0533 to −0.0178)	0.0148 (−0.0136 to 0.0432)	0.0090 (−0.0259 to 0.0438)
**Longitudinal**				
Continuous FGCRS × time	−0.0004^†^ (−0.0008 to −0.0001)	−0.0008^†^ (−0.0012 to −0.0004)	−0.0005^†^ (−0.0009 to −0.0000)	−0.0006^†^ (−0.0011 to −0.0001)
**FGCRS categories**				
Lowest risk × time	Reference	Reference	Reference	Reference
Middle risk × time	−0.0025 (−0.0056 to 0.0005)	−0.0048^†^ (−0.0084 to −0.0011)	−0.0038^†^ (−0.0076 to −0.0000)	−0.0030 (−0.0075 to 0.0015)
Highest risk × time	−0.0038^†^ (−0.0069 to −0.0008)	−0.0056^†^ (−0.0093 to −0.0020)	−0.0039^†^ (−0.0077 to −0.0001)	−0.0053^†^ (−0.0098 to −0.0008)

* *Adjusted for education, body mass index, alcohol consumption, physical activity, social activity, baseline number of cardiovascular diseases, and baseline global cognition score.*

† *P < 0.05*.

Over the 22 years of follow-up (median duration: 5.3 years; interquartile range: 2.9–9.0 years), in multi-adjusted mixed-effects models, increased FGCRS as continues variable was dose-dependently related to global motor function (β = −0.0004; 95% CI: −0.0008 to −0.0001), dexterity (β = −0.0008; 95% CI: −0.0012 to −0.0004), gait (β = −0.0005; 95% CI: −0.0009 to −0.0000), and hand strength (β = −0.0006; 95% CI: −0.0011 to −0.0001) decline, respectively. β denoted the rate of accelerated decline in motor function per 1-score change in FGCRS per year.

**Figure 1:**
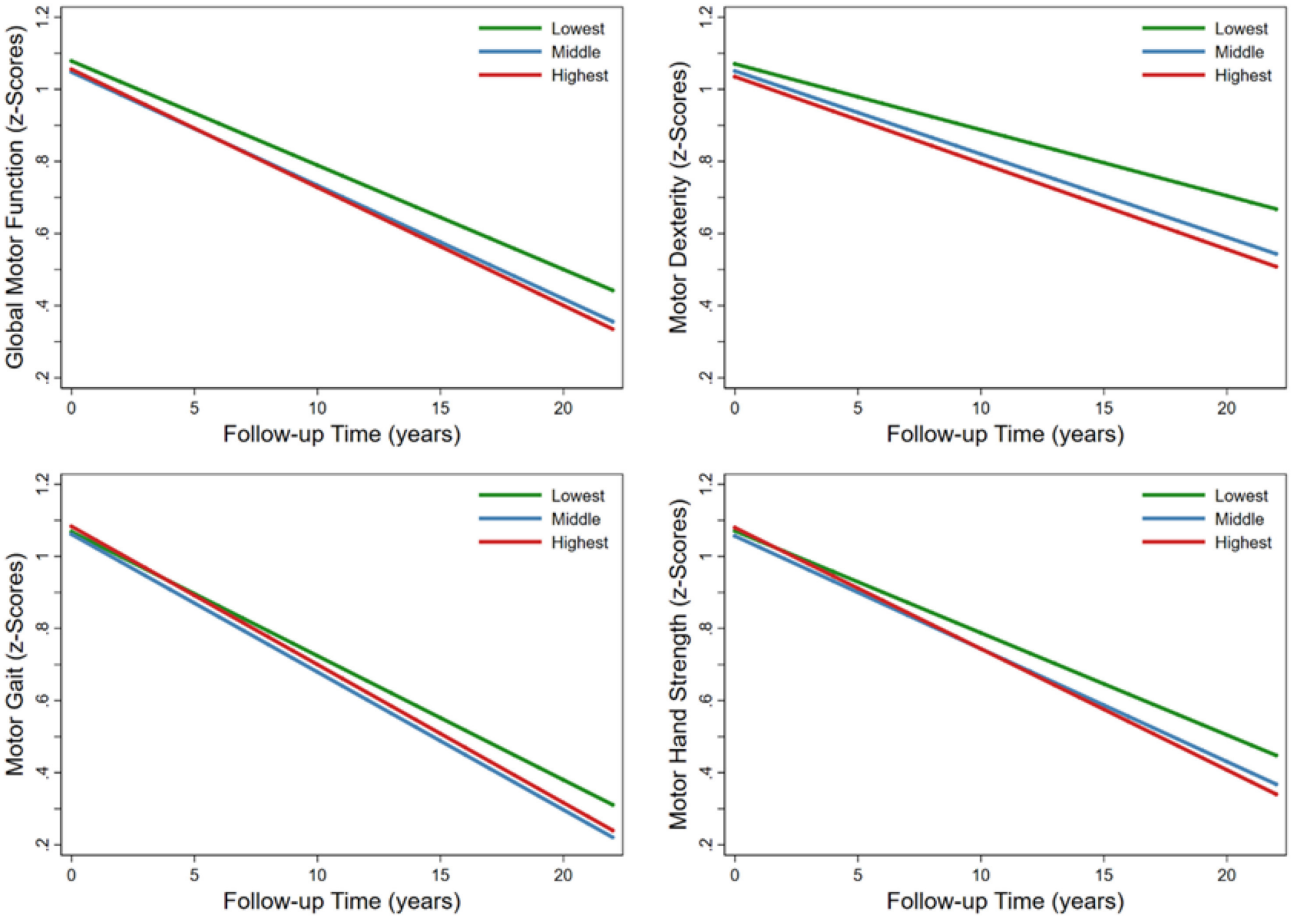
Trajectories of average annual changes in global and specific motor functions by Framingham General Cardiovascular Risk Score (FGCRS) in tertiles. Model was adjusted for education, body mass index, alcohol consumption, physical activity, social activity, baseline number of cardiovascular diseases, and baseline global cognition score.

Compared with the lowest FGCRS, the highest FGCRS was associated with faster decline in global motor function (β = −0.0038; 95% CI: −0.0069 to −0.0008), dexterity (β = −0.0056; 95% CI: −0.0093 to −0.0020), gait (β = −0.0039; 95% CI: −0.0077 to −0.0001), and hand strength (β = −0.0053; 95% CI: −0.0098 to −0.0008) over the follow-up time (Table 2; Figure 1).

### The Mediating Role of CVDs and Global Cognitive Function Changes

**Figure 2:**
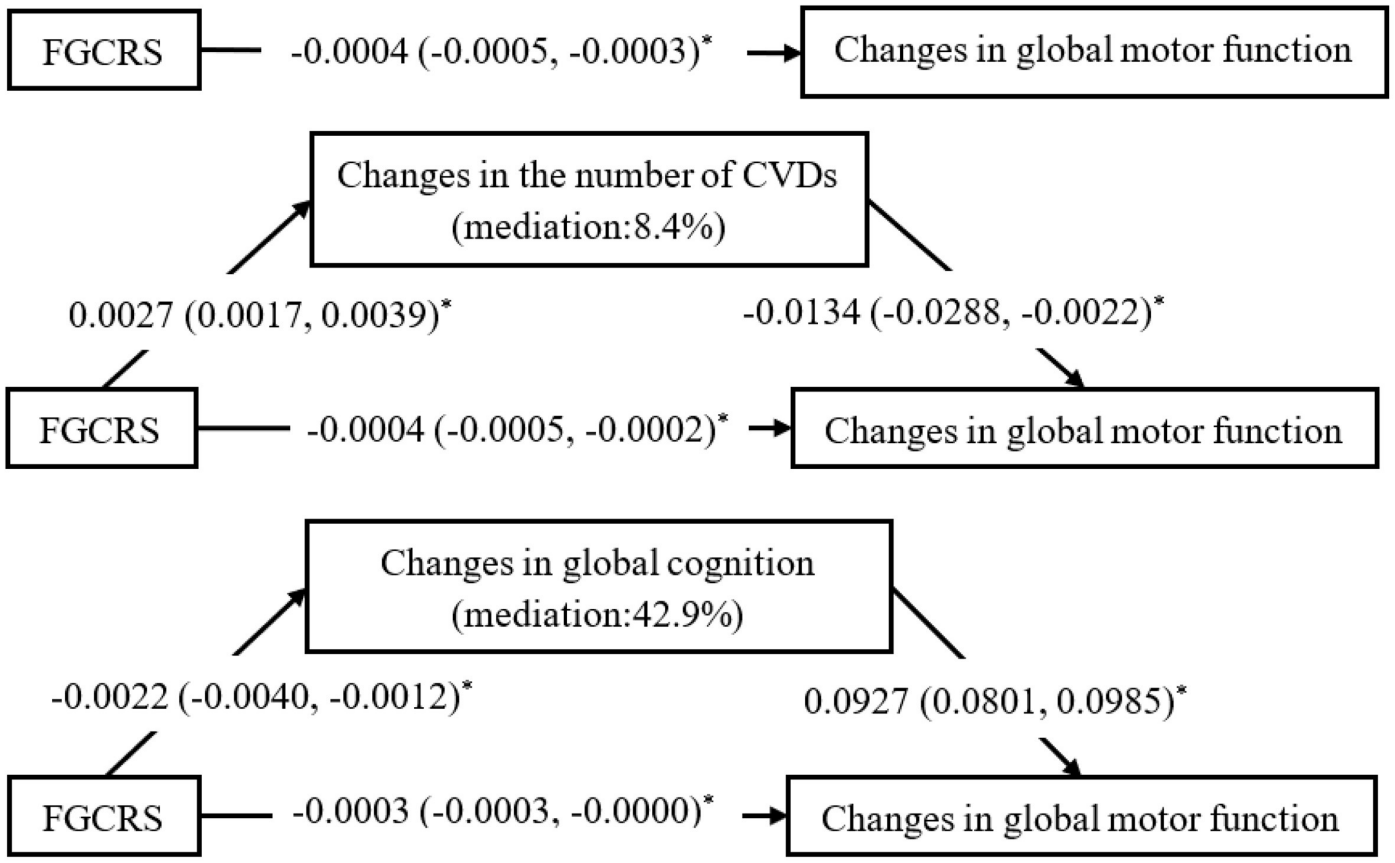
Mediating effects of changes in the number of cardiovascular diseases (CVDs) and global cognitive function on the association between Framingham General Cardiovascular Risk Score (FGCRS) and global motor function changes. β-coefficients and 95% confidence intervals were calculated using bias-corrected bootstrapping. Mediation model adjusted for education, body mass index, alcohol consumption, physical activity, social activity, baseline number of cardiovascular diseases, and baseline global cognition score. *P < 0.05.

In the mediation analysis, the direct effect of FGCRS on changes in global motor function was diminished when the changes in the number of CVDs (β =−0.0004; 95% CI: −0.0005 to −0.0002) and global cognition (β =−0.0003; 95% CI: −0.0003 to −0.0000) were added to the model separately. In mediating effect models, 8.4% of the association between FGCRS and global motor function was mediated by CVDs accumulation, and 42.9% was mediated by changes in cognitive function during follow-up (Figure 2).

### Sensitivity Analysis

Similar results to the initial analysis were obtained in the linear mixed-effects models and mediation analysis when we excluded 382 participants with dementia at baseline and during the follow-up period (Supplementary Table 3 and Supplementary Figure 2). Furthermore, the results were not materially altered after further adjusted depression (Supplementary Table 4).

## Discussion

In this prospective community-based cohort study, we found that (1) higher FGCRS is associated with a faster decline in global motor function, dexterity, gait, and hand strength over time; and (2) CVD accumulation (about 8.4%) and cognitive decline (about 42.9%) partially mediated the association between FGCRS and motor function decline.

The relationship between single cardiovascular risk factors (e.g., older age, lower HDL-C levels, diabetes mellitus, and antihypertensive medications use) and motor function decline has been reported in many studies (30–33). As cardiovascular risk factors tend to occur in clusters, FGCRS combines vascular risk factors by assigning values to various risk factors with different severity levels synthetically as a global measure of vascular risk burden which may better predict the risk of future CVDs (9). To the best of our knowledge, only two studies have investigated the relationship between FGCRS and walking speed or grip strength. One of them showed that higher FGCRS was associated with a greater risk of subsequent walking speed limitation (10). In another study, participants with higher FGCRS performed poorer in walking speed, but FGCRS was not related to grip strength and finger tapping (11). In this study, for the first time, we used a comprehensive composite measure of global motor function including motor dexterity, gait, and hand strength to reduce random variability by converting the raw scores of 10 tests. We found that higher FGCRS was related to a faster decline in global motor function, dexterity, gait, and hand strength over time.

From the perspective of life course epidemiology, one type of the chains of risk models is that each exposure not only has an independent impact on the risk of diseases but also increases the risk of subsequent exposures leading to changes in health outcomes (34). It has been known that higher FGCRS may increase the risk of CVDs (35, 36) and cognitive decline (37–39), and both conditions have been related to an accelerated rate of decline in motor function (40–43), whereas, to the best of our knowledge, no studies to date have assessed the possible mediating effects of CVDs accumulation or cognitive decline in the relationship between FGCRS and motor function trajectories. In the mediation analysis, we found that the mediation effect of cognitive function changes on the FGCRS-motor function association seems stronger than that of CVDs. Possible explanations for the difference could be the following: (1) the effect of cognitive function on motor function might be stronger than that of CVD indeed; and (2) it is possible that a mild form of CVD was included in the analysis due to the fact that those with severe/acute CVD died during the follow-up, and motor function could not be assessed in these participants (i.e., selective survival). Thus, further population-based longitudinal studies are warranted to compare the mediating effect of cognitive function and CVDs on the vascular risk burden-motor function association. Our findings highlight the need to control CVDs and monitor cognitive function in older adults with a high vascular risk burden for the prevention of physical function decline.

There are two pathways that may explain the link between cardiovascular risk burden and motor function decline. First, cardiovascular risk factors (e.g., smoking, diabetes, and high cholesterol) are traditional risk factors for atherosclerosis, which is a major component of cardiovascular diseases (44). Atherosclerosis involves multiple inflammatory cytokines that may affect muscle metabolism by altering blood vessel dynamics and cause skeletal muscle breakdown in turn (45). Meanwhile, skeletal muscle is a major site of glucose metabolism by insulin, and the insulin resistance accompanying atherosclerosis might link to a decrease in muscle mass (46). In addition, increased atherosclerotic plaques narrow the arteries and reduce muscle perfusion leading to mobility impairment (47). Second, exposure to cardiovascular risk factors accelerates structural brain aging (e.g., white matter hyperintensity and brain atrophy) and cognitive decline (48), which may affect gait and mobility by several cognitive domains including visuospatial abilities, executive-attentional function, and memory resources (49). Moreover, the changes in composite indicators of abstract thinking and verbal expression were independently related to the decline in motor function (50).

As a community-based longitudinal cohort study, this study presents several strengths. We followed participants with a relatively large sample size for up to 22 years annually. Furthermore, we assessed cardiovascular risk burden by FGCRS as exposure and evaluated comprehensibly global motor function and its subcomponents by a battery of 10 tests over the yearly follow-up. However, some limitations need to be pointed out. First, participants were recruited from the community as volunteers with generally good education and performed well on cognitive tests; thus, the representativeness of the findings was limited, and the observed associations were likely to be underestimated. Moreover, the study participants were in good living conditions with regular medical supervision, and thus, caution is needed when generalizing our findings to other populations. Second, CVD events were assessed based on retrospective self-reports, which may have contributed to information bias leading to underestimation of the given association. Third, information on specific medications used for the treatment of CVD was not available, and thus, the role of CVD-related drug use in the association between FGCRS and motor functions could not be assessed. Finally, potential confounders such as occupational characteristics and medical histories of brain trauma could not be considered due to data.

In conclusion, this study provides evidence of the association between cardiovascular risk burden assessed by the FGCRS and trajectories of mobility and hand strength in aging. The accumulation of CVDs and cognitive decline may partially mediate the relationship between cardiovascular risk burden and changes in motor function. Our findings emphasize the importance of monitoring and controlling cardiovascular risk to prevent both CVDs and cognitive decline in order to slow down the progression of motor impairments and disability among older adults.

## Data Availability Statement

The datasets presented in this study can be found in online repositories. The names of the repository/repositories and accession number(s) can be found below: The Rush Alzheimer's Disease Center Resource Sharing Hub, https://www.radc.rush.edu.

## Ethics Statement

The studies involving human participants were reviewed and approved by Rush University Medical Center's Institutional Review Board. The patients/participants provided their written informed consent to participate in this study.

## Author Contributions

ZW and KC: performed statistical analysis, drafting the article. RS and XL: analysis and interpretation of data. XQ and AB: revising the article critically for important intellectual content. DB and WX: conception and design, major role in the acquisition of data, final approval of the version to be published. All authors contributed to the article and approved the submitted version.

## Funding

WX received grants from the Swedish Research Council (Nos. 2017-00981 and 2021-01647), the Swedish Council for Health Working Life and Welfare (No. 2021-01826), the National Natural Science Foundation of China (No. 81771519), Alzheimerfonden (No. 2021-2022), Karolinska Institutet Research Foundation (No. 2020-01660), Lindhés Advokatbyrå AB (No. 2021-0134), Stiftelsen För Gamla Tjänarinnor (No. 2021-2022) and Demensfonden. Bennett received grants from the National Institutes of Health (R01AG17917 and UH2NS100599).

## Conflict of Interest

The authors declare that the research was conducted in the absence of any commercial or financial relationships that could be construed as a potential conflict of interest.

## Publisher's Note

All claims expressed in this article are solely those of the authors and do not necessarily represent those of their affiliated organizations, or those of the publisher, the editors and the reviewers. Any product that may be evaluated in this article, or claim that may be made by its manufacturer, is not guaranteed or endorsed by the publisher.

## Abbreviations

ADL: activities of daily living
BMI: body mass index
CI: confidence interval
CVD: cardiovascular disease
DBP: diastolic blood pressure
FGCRS: Framingham General Cardiovascular Risk Score
HDL: high-density lipoprotein
MAP: Rush Memory and Aging Project
MMSE: Mini-Mental State Examination
SBP: systolic blood pressure
TC: total cholesterol.
