# Supplementary material for: Influence of Cardiovascular Risk Burden on Motor Function Among Older Adults: Mediating Role of Cardiovascular Diseases Accumulation and Cognitive Decline
Source: Front Med (Lausanne). 2022 Apr 26;9:856260. doi: 10.3389/fmed.2022.856260 (PMC9087801; doi:10.3389/fmed.2022.856260)
Supplement: Supplementary file 1 [file Data_Sheet_1.docx]

***Supplementary Material***

**Supplementary Table 1.** Framingham General Cardiovascular Risk Score (FGCRS) Calculating for Women.

**Supplementary Table 2.** Framingham General Cardiovascular Risk Score (FGCRS) Calculating for Men.

**Supplementary Table 3.** β-coefficients and 95% confidence intervals (CIs) for the association of the Framingham General Cardiovascular Risk Score (FGCRS) with the changes of global and specific motor functions over time in participants without dementia at baseline and during the follow-up period (n=1016): results from Mixed effect models ^a^.

**Supplementary Table 4**. β-coefficients and 95% confidence intervals (CIs) for the association of the Framingham General Cardiovascular Risk Score (FGCRS) with the changes of global and specific motor functions over time further adjusted depression: results from Mixed effect models^*^.

**Supplementary Figure 1.** Flowchart of study participants.

**Supplementary Figure 2**. Mediating effects of changes in the number of cardiovascular diseases (CVDs) and global cognitive function on the association between Framingham General Cardiovascular Risk Score (FGCRS) and global motor function changes in participants without dementia at baseline and during the follow-up period (n=1016).

**Supplementary Table 1.** Framingham General Cardiovascular Risk Score (FGCRS) Calculating for Women.

| **Points** | **Age (years)** | **HDL (mg/dl)** | **TC (mg/dl)** | **SBP Not Treated (mm Hg)** | **SBP Treated (mm Hg)** | **Smoker** | **Diabetic** |  |
| --- | --- | --- | --- | --- | --- | --- | --- | --- |
| -3 |  |  |  | <120 |  |  |  |  |
| -2 |  | 60+ |  |  |  |  |  |  |
| -1 |  | 50-59 |  |  | <120 |  |  |  |
| 0 | 30-34 | 45-49 | <160 | 120-129 |  | No | No |  |
| 1 |  | 35-44 | 160-199 | 130-139 |  |  |  |  |
| 2 | 35-39 | <35 |  | 140-149 | 120-129 |  |  |  |
| 3 |  |  | 200-239 |  | 130-139 | Yes |  |  |
| 4 | 40-44 |  | 240-279 | 150-159 |  |  | Yes |  |
| 5 | 45-49 |  | 280+ | 160+ | 140-149 |  |  |  |
| 6 |  |  |  |  | 150-159 |  |  |  |
| 7 | 50-54 |  |  |  | 160+ |  |  |  |
| 8 | 55-59 |  |  |  |  |  |  |  |
| 9 | 60-64 |  |  |  |  |  |  |  |
| 10 | 65-69 |  |  |  |  |  |  |  |
| 11 | 70-74 |  |  |  |  |  |  |  |
| 12 | 75+ |  |  |  |  |  |  |  |
| *Points allotted* |  |  |  |  |  |  |  | *Total* |

Abbreviations: HDL= High-Density Lipoprotein, SBP= Systolic Blood Pressure, TC= Total Cholesterol.

**Supplementary Table 2.** Framingham General Cardiovascular Risk Score (FGCRS) Calculating for Men.

| **Points** | **Age (years)** | **HDL (mg/dl)** | **TC (mg/dl)** | **SBP Not Treated (mm Hg)** | **SBP Treated (mm Hg)** | **Smoker** | **Diabetic** |  |
| --- | --- | --- | --- | --- | --- | --- | --- | --- |
| -2 |  | 60+ |  | <120 |  |  |  |  |
| -1 |  | 50-59 |  |  |  |  |  |  |
| 0 | 30-34 | 45-49 | <160 | 120-129 | <120 | No | No |  |
| 1 |  | 35-44 | 160-199 | 130-139 |  |  |  |  |
| 2 | 35-39 | <35 | 200-239 | 140-159 | 120-129 |  |  |  |
| 3 |  |  | 240-279 | 160+ | 130-139 |  | Yes |  |
| 4 |  |  | 280+ |  | 140-159 | Yes |  |  |
| 5 | 40-44 |  |  |  | 160+ |  |  |  |
| 6 | 45-49 |  |  |  |  |  |  |  |
| 7 |  |  |  |  |  |  |  |  |
| 8 | 50-54 |  |  |  |  |  |  |  |
| 9 |  |  |  |  |  |  |  |  |
| 10 | 55-59 |  |  |  |  |  |  |  |
| 11 | 60-64 |  |  |  |  |  |  |  |
| 12 | 65-69 |  |  |  |  |  |  |  |
| 13 |  |  |  |  |  |  |  |  |
| 14 | 70-74 |  |  |  |  |  |  |  |
| 15 | 75+ |  |  |  |  |  |  |  |
| *Points allotted* |  |  |  |  |  |  |  | *Total* |

Abbreviations: HDL= High-Density Lipoprotein, SBP=Systolic Blood Pressure, TC= Total Cholesterol.

**Supplementary Table 3.** β-coefficients and 95% confidence intervals (CIs) for the association of the Framingham General Cardiovascular Risk Score (FGCRS) with the changes of global and specific motor functions over time in participants without dementia at baseline and during the follow-up period (n=1016): results from Mixed effect models ^a^.

| Cardiovascular Disease Risk | Global Motor Function | Motor Dexterity | Motor Gait | Motor Hand Strength |
| --- | --- | --- | --- | --- |
| Baseline |  |  |  |  |
| Continuous FGCRS | -0.0065^*^ (-0.0097 to -0.0033) | -0.0056^*^ (-0.0078 to -0.0034) | -0.0007 (-0.0044 to 0.0030) | -0.0040 (-0.0088 to 0.0007) |
| FGCRS categories |  |  |  |  |
| Lowest risk | Reference | Reference | Reference | Reference |
| Middle risk | -0.0421^*^ (-0.0705 to -0.0138) | -0.0305^*^ (-0.0501 to -0.0108) | -0.0075 (-0.0403 to 0.0254) | -0.0360 (-0.0780 to 0.0060) |
| Highest risk | -0.0315^*^ (-0.0602 to -0.0028) | -0.0384^*^ (-0.0583 to -0.0185) | 0.0112 (-0.0220 to 0.0445) | -0.0091 (-0.0517 to 0.0334) |
| Longitudinal |  |  |  |  |
| Continuous FGCRS $\boldsymbol{\times}$ time | -0.0005^*^ (-0.0009 to -0.0002) | -0.0008^*^ (-0.0012 to -0.0005) | -0.0005^*^ (-0.0010 to -0.0000) | -0.0004 (-0.0010 to 0.0002) |
| FGCRS categories |  |  |  |  |
| Lowest risk $\boldsymbol{\times}$ time | Reference | Reference | Reference | Reference |
| Middle risk $\boldsymbol{\times}$ time | -0.0032 (-0.0064 to 0.0001) | -0.0046^*^ (-0.0078 to -0.0013) | -0.0060^*^ (-0.0102 to -0.0017) | -0.0010 (-0.0059 to 0.0040) |
| Highest risk $\boldsymbol{\times}$ time | -0.0055^*^ (-0.0088 to -0.0022) | -0.0059^*^ (-0.0092 to -0.0026) | -0.0049^*^ (-0.0092 to -0.0005) | -0.0043 (-0.0094 to 0.0009) |

^a^ Adjusted for education, body mass index, alcohol consumption, physical activity, social activity, baseline number of cardiovascular diseases and baseline global cognition score.

^*^ *P* < 0.05.

**Supplementary Table 4.** β-coefficients and 95% confidence intervals (CIs) for the association of the Framingham General Cardiovascular Risk Score (FGCRS) with the changes of global and specific motor functions over time further adjusted depression: results from Mixed effect models^*^.

| Cardiovascular Disease Risk | Global Motor Function | Motor Dexterity | Motor Gait | Motor Hand Strength |
| --- | --- | --- | --- | --- |
| Baseline |  |  |  |  |
| Continuous FGCRS | -0.0048^†^ (-0.0076 to -0.0020) | -0.0050^†^ (-0.0070 to -0.0030) | 0.0001 (-0.0032 to 0.0033) | -0.0012 (-0.0052 to 0.0027) |
| FGCRS categories |  |  |  |  |
| Lowest risk | Reference | Reference | Reference | Reference |
| Middle risk | -0.0315^†^ (-0.0559 to -0.0070) | -0.0203^†^ (-0.0379 to -0.0026) | -0.0065 (-0.0349 to 0.0218) | -0.0175 (-0.0520 to 0.0170) |
| Highest risk | -0.0235 (-0.0481 to 0.0012) | -0.0361^†^ (-0.0539 to -0.0183) | 0.0145 (-0.0140 to 0.0430) | 0.0075 (-0.0273 to 0.0423) |
| Longitudinal |  |  |  |  |
| Continuous FGCRS $\boldsymbol{\times}$ time | -0.0004^†^ (-0.0008 to -0.0001) | -0.0008^†^ (-0.0012 to -0.0004) | -0.0004^†^ (-0.0009 to -0.0000) | -0.0006^†^ (-0.0011 to -0.0001) |
| FGCRS categories |  |  |  |  |
| Lowest risk $\boldsymbol{\times}$ time | Reference | Reference | Reference | Reference |
| Middle risk $\boldsymbol{\times}$ time | -0.0027 (-0.0057 to 0.0003) | -0.0051^†^ (-0.0087 to -0.0014) | -0.0040^†^ (-0.0078 to -0.0003) | -0.0030 (-0.0075 to 0.0015) |
| Highest risk $\boldsymbol{\times}$ time | -0.0038^†^ (-0.0068 to -0.0008) | -0.0058^†^ (-0.0094 to -0.0021) | -0.0038^†^ (-0.0076 to -0.0000) | -0.0054^†^ (-0.0100 to -0.0008) |

^*^Adjusted for education, body mass index, alcohol consumption, physical activity, social activity, depression, baseline number of cardiovascular diseases and baseline global cognition score.

^†^*P* < 0.05.

Baseline participants, n=2192

573 with missing FGCRS, 260 with disability at baseline.

462 with missing data on global motor function.

Participants in analysis, n=1378

Lowest FGCRS risk

n=530 (38.5%)

Highest FGCRS risk

n=436 (31.6%)

Middle FGCRS risk

n=412 (29.9%)

**Supplementary Figure 1.** Flowchart of study participants.

The participants were categorized into lowest (4<=FGCRS<=14), middle (14<FGCRS<=17), and highest (17<FGCRS<=28) risk groups based on the FGCRS tertiles.

Abbreviations: FGCRS, Framingham General Cardiovascular Risk Score.


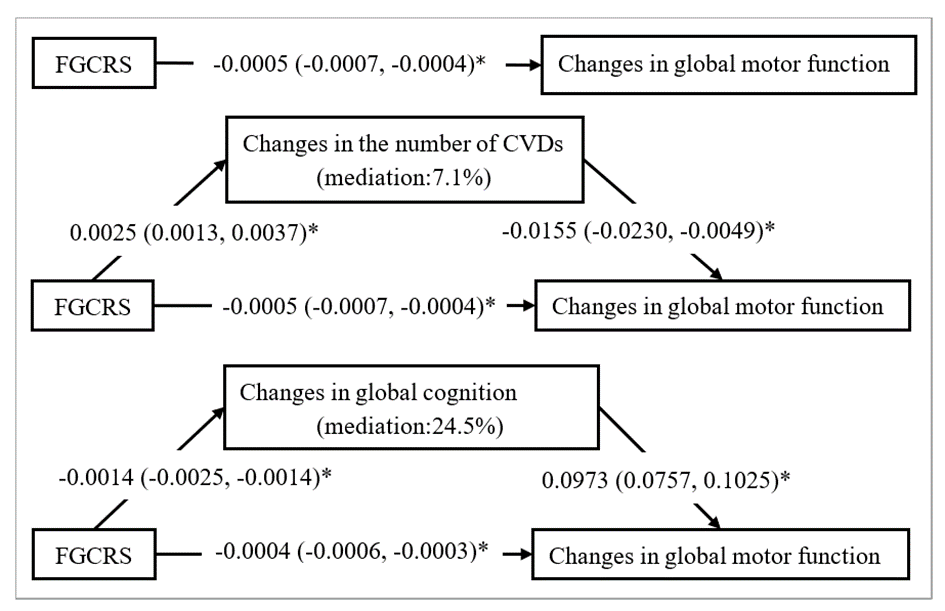


**Supplementary Figure 2**. Mediating effects of changes in the number of cardiovascular diseases (CVDs) and global cognitive function on the association between Framingham General Cardiovascular Risk Score (FGCRS) and global motor function changes in participants without dementia at baseline and during the follow-up period (n=1016).

β-coefficients and 95% confidence intervals were calculated using bias-corrected bootstrapping.

Mediation model adjusted for education, body mass index, alcohol consumption, physical activity, social activity, baseline number of cardiovascular diseases and baseline global cognition.

^*^ *P* < 0.05.
